# Mental health among single mothers in Cyprus: a cross-sectional descriptive correlational study

**DOI:** 10.1186/s12905-019-0763-9

**Published:** 2019-05-16

**Authors:** Elena Rousou, Christiana Kouta, Nicos Middleton, Maria Karanikola

**Affiliations:** Department of Nursing, School of Health Sciences, Cyprus University of Technology, 15, Vragadinou str, 3041 Limassol, Cyprus

**Keywords:** Single parent family, Single mothers, Health, Psychological distress, Social support

## Abstract

**Background:**

International literature reveals that single mothers experience increased levels of chronic stress, which is mainly due to economic hardship and reduced levels of social support. Eventually this leads to psychological distress. While most of the studies commonly identify that mental health disorders are common among single mothers compared to their married counterparts, the magnitude of the problem might be even larger since diagnosis-specific tools may mask important levels of distress of milder intensity. This study aims to assess the level of mental distress experienced by single mothers as measured by the GHQ-28, and how it is influenced by socioeconomic factors, as well as the level of perceived social support.

**Methods:**

Between January and March 2012, Greek speaking single mothers who reside in Cyprus were recruited by either personal conduct through Single Mothers’ Association (SMA), or by using snowball sampling technic. Mental distress was assessed with the General Health Questionnaire (GHQ-28) and perceived social support with the Social Provision Scale (SPS). All scales were completed anonymously and voluntarily by 316 single mothers. Univariable and multivariable associations with socio-demographic characteristics were investigated using chi-square tests and in multivariable backward stepwise logistic regression models respectively. Odds ratio of psychological distress across decreasing levels of social support were estimated in logistic regression models. .

**Results:**

As many as 44.6% of the sample appeared to experience psychological distress (GHQ-28 total score ≥ 5). Strong associations with all health assessment tools were observed with variables relating to the lowest monthly family income, the presence of economic difficulties, the higher educational level, the age group 35–44 years and pre-existing illness. Social support as perceived by the mothers displayed a strong negative independent association with psychological distress, even after adjusting confounders.

**Conclusion:**

This study highlights that single mothers are very likely to experience poor psychological well-being. With a steady rise in the proportion of single-parent families headed by a mother, these findings highlight a significant issue that would adversely affect many women and consequently their children and the community. It also emphasizes the necessity for interventions and strategies at community level in order to support this vulnerable population group.

## Background

Family kin networks have been identified as important determinants of mental well-being. In particular, single motherhood is related to poor mental health [1–4]. Single mothers demonstrate higher levels of psychiatric symptomatology, e.g. depressive symptoms compared to other female groups. Poor mental health in single mothers is associated with financial insecurity and increased frequency of daily stressors [5]. Other data show that access to support networks is essential in empowering people cope with every-day problems and manage challenges [6, 7]. Studies has shown that higher levels of mastery and social support were found to be associated with less depressive symptoms [8], and that single mothers without additional personal support for their child, younger, as well as poor single mothers showed higher values of psychological distress [9].

Similarly, literature review supports that single motherhood places women not only in adverse social position, but also in vulnerability regarding their overall health status mainly due to exposure to conditions of prolonged stress compared to partnered mothers [2]. Moreover, a growing body of evidence suggests that single motherhood is associated with risky health-related behaviors (e.g. smoking, alcohol), minor psychiatric morbidity, psychological distress, psychosomatic symptoms and depression [3, 4, 10]. To date, there are no studies on the prevalence of mental distress among single mothers in Cyprus.

While studies commonly identify that mental health disorders are increased among single mothers compared to their married counterparts, the magnitude of the problem might be even larger since diagnosis-specific tools may mask important levels of distress of milder intensity [11–27].

Fewer studies have used more generic non-diagnosis specific tools in order to assess the overall level of health, quality of life or psychological well-being among single mothers [9, 16, 20, 28–30].

The GHQ-28, is one such generic tool of mental distress, which has been extensively used is several studies of clinical or community samples, but it has not been used in the case of single mothers. The GHQ can be used to estimate the prevalence of mental distress and is designed to assess an individual’s ability to carry out normal ‘healthy’ functions, rather than more severe disorders such as psychotic depression or acute schizophrenia [31]. A number of studies have found it to be a valid method of case identification compared with clinical interview [32, 33].

The aim of this study is to add to the existing literature by assessing level of mental distress experienced among single mothers in Cyprus as measured by the GHQ-28 and explore the extent to which mental distress in this vulnerable population group varies in terms of socioeconomic factors and disadvantage, as well as levels of perceived social support.

## Method

### Study population and design

A cross-sectional descriptive correlational study was conducted. Between January and March 2012, we recruited a total sample of 315 single mothers to participate in this study. Regarding eligibility criteria, the target population included Greek speaking single mothers from all over Cyprus, irrespective of whether divorced, separated, widows or unmarried), who had the custody of at least one child under the age of 18. Through the Single Mothers Association (SMA) we recruited 179 single mothers. Single mothers were approached by the researcher face to face when they visited the SMA, explained the purpose of the study and their participation was voluntarily. Nearly, all single mothers approached were willing and participate in the study. In parallel, through snowball sampling technique among those who agreed to participate, we reached another 136 single mothers. These two methods of data collection gave us the opportunity to achieve a more heterogeneous sample, as the socio-demographic characteristics of single mothers may differ between these two groups. Sample size calculations were based on precision analysis with finite population correction considering the latest census estimate of around 17,000 single parent families headed by the mother in Cyprus. It was estimated that a minimum sample of 300 participants was required to achieve a precision of ±5 percentile points with 95% confidence around an expected prevalence estimate of mental distress around 30% in this population group based on previous literature [19].

After obtaining informed consent, the questionnaire pack was distributed to the participants in person and completed questionnaires were returned in sealed envelopes. Approval for this study has been obtained, both by the National Bioethics committee (EEBK, 2011.01.42), and the Ethics committee of the Cyprus University of Technology.

### Instruments

#### General health questionnaire (28 item) GHQ 28

The General Health Questionnaire (GHQ) is a screening tool used to assess the current mental wellbeing in primary care settings. Rather than giving a specific psychiatric diagnosis, the GHQ assess normal ‘healthy’ functioning and the development of new, distressing symptoms [31]. The GHQ-28 was derived by means of factor analysis from the original 60-item 4-point scale GHQ. Its four subscales, ‘somatic symptoms’, ‘anxiety/insomnia’, ‘social dysfunction’ and ‘severe depression’ are designed to give more detailed information than a single severity score [34].

These four subscales seem to apply for single mothers’ population, as it includes the basic variables that based on the literature they experience in a higher extent as a result of their family situation [35]. Therefore, GHQ-28 seems an appropriate tool to assess single mothers’ mental well-being as what places single mothers into an adverse social position is the prolonged stress mainly due to unemployment, financial hardship, social exclusion and childrearing responsibilities [2, 36, 37].

In the context of this study, among the several ways of scoring, the traditional method (binary code-0,0,1,1) has been used [38]. According to parameters such as particular version, scoring method and type of population, the threshold or cut-scores for the GHQ vary, ranging from 3/4 to 7/8 on the GHQ-28 [33]. However, when screening in primary care setting, using community samples, lower threshold scores can be used. In the case of this study, we used as cut-score the 5/6 as it seemed more appropriate [39].

The GHQ-28 has been used extensively worldwide in both primary care as well as community populations, both in research as well as in clinical practice, since it is considered one of the most robust screening tools available to assess psychological well-being and detect possible psychiatric morbidity [40]. It has been translated into numerous languages and cross-culturally validated in adult populations. It has been used in Greek-speaking population with a Chronbach’s a = .93 [41], and therefore we used their Greek language version after obtaining the necessary approvals for its use by GL Assessment Ltd.

#### Social provision scale (SPS)

To assess the perceived social support among single mothers, the Social Provision Scale, developed by Cutrona and Russel [42], was used. It measures the perceived support provided to a person through their social relationships with others. The SPS is a 4-point scale that includes 24 items and a theoretical range from 24 to 96. Characteristic statements from the scale include: “There are people I can depend on to help me if I really need it”, or “I feel part of a group of people who share my attitudes and beliefs”. Overall, the scale is structured so that items tap on six types of social support provision and a global social support score can be calculated [42, 43].

SPS scale was forward and backward translated by two bilingual academics -a sociologist and a psychologist – from English to Greek in order to retain equivalence based on recommended guidelines [44]. A pilot study with the final version of the instrument prior the main study has been conducted in a group of 23 single mothers and minor correction were made based on comments related to its general comprehensibility.

A number of studies have been conducted with the use of this scale with a variety of community samples [23, 45–47], and its validity and reliability as well as the instruments’ factor structure has been well supported [42]. In the original study, the Cronbach’s alpha coefficient the total Social Provision scores was reported to be 0.915 [43], and in the present study, was 0.92.

### Data analysis

Descriptive and inferential statistical analyses were undertaken to examine the socio-demographic characteristics of the participants and explore the relationship between the variables of interest. Socio-demographic characteristics, level of mental distress and perceived social provision among the participants were expressed in either absolute and relative frequencies or mean values and standard deviation as appropriate.

The GHQ − 28 has a 4-item response scale anchored with ‘not at all’, ‘no more than usual’, ‘Rather more than usual’, and ‘Much more than usual’. Several scoring options are available; it has been identified that the best thresholds in terms of specificity and sensitivity to determine cases in community samples was 5/6 using the GHQ method [39], and therefore this was adopted in this study. In parallel, the Likert method was used to indicate the symptoms severity on the four subscales theoretical range 0–21) as per recommended method for assessment of the subscales [48].

In the absence of cut-off points based on a theoretical criterion, quartiles of the overall SPS score were used to characterize participants in terms of the level of perceived social support.

The extent to which psychological distress differed according to the socio-demographic characteristics of the participants was investigated in a series of chi-square tests. Furthermore, associations with socio-demographic variables were explored in multivariable backward stepwise logistic regression models. Odds ratio (and 95% confidence intervals) of mental distress across quartiles of participants with decreasing levels of social support were estimated in logistic regression models before and after adjusting for the potential confounding effect of socio-demographic variables. Fully adjusted models included all of the socio-demographic variables. The Statistical Package for Social Science (SPSS) Program version 17.0 was used in analyzing the data.

## Results

### Socio-demographic characteristics of single mothers

Socio-demographic characteristics of the sample are summarized in Table 1. Single mothers that participated in this study had a mean age of 39.2 years (SD = 6.64, range: 23–59). Regarding family status, 73.1% were divorced, and as many as half were the head of a single parent family for more than 5 years (46.5%). As for the number of dependent children, the majority had the custody of either one (40.3%) or two (43.5%) children, of whom the majority were under the age of 12 years (64.9%).

**Table 1:** Socio-demographic characteristics of the participants and prevalence of clinically significant psychological distress (GHQ-28 ≥ 5)

Demographic characteristics		GHQ-28 (total score) ≥ 5			
	% (*n*=)	%	x^2^	Df	*P* value
Age					
*23–34*	25,4 (80)	40.5	6.417	2	**0.040**
*35–44*	51,7 (163)	57.1			
*45–59*	22,9 (72)	46.5			
Educational level					
*Junior high school*	7,9 (25)	60	10.290	3	**0.016**
*High school*	32,4 (102)	49.5			
*Higher education*	23.5 (74)	63.5			
*University*	36.2 (114)	40.7			
Single family status					
*Separated*	9,5 (30)	53.3	1.768	3	0.622
*Widow*	9,2 (29)	60.7			
*Divorced*	73,1 (231)	49.6			
*Unmarried*	7,9 (25)	44			
How long as single mother					
*< 2 years*	20.5 (65)	62.5	5.613	2	0.060
*2–4 years*	33 (103)	43.7			
*5 or more years*	46.5 (147)	50.5			
Children age < 12 years old					
*Yes*	64.9 (204)	49.5	0.302	1	0.583
*No*	35.1 (109)	52.8			
Employment status					
*Economically inactive*	8.2 (26)	69.2	6.792	3	0.079
*Unemployed*	7.6 (24)	50			
*Working part-time*	10.4 (33)	62.5			
*Working full-time*	73.4 (232)	46.8			
Monthly family income					
*> 1000*	27.8 (88)	65.5	15.297	5	**0.009**
*1000–1499*	27.2 (86)	50			
*1500–1999*	18.0 (57)	42.9			
*2000–2499*	10.1 (32)	40.6			
*2500–3499*	12.0 (38)	47.4			
*≥ 3500*	4.4 (14)	21.4			
Member of SMA					
*No*	56.8 (179)	46.4	2.598	1	0.107
*Yes*	43.2 (136)	55.6			
Single mothers’ allowance recipient					
*Yes*	58.9 (186)	53.3	1.382	1	0.240
*No*	40.8 (129)	46.5			
Financial child support from the father					
*Yes*	51.9 (163)	45.4	3.512	1	0.061
*No*	48.1 (152)	56			
Economic hardship during the last 12 months					
*Yes*	79.7 (252)	57.2	23.235	1	**< 0.001**
*No*	20.3 (64)	23.4			
Limiting-long standing illness					
*Yes*	49.4 (156)	65.2	26.976	1	**< 0.001**
*No*	50.6 (160)	35.8			
Physical activity					
*Yes*	30.8 (97)	56.4	10.626	1	**0.001**
*No*	69.2 (218)	36.5			
Smoking habit					
*Yes*	39.4 (124)	53.2	0.693	1	0.405
*No*	60.6 (191)	48.4			
Alcohol consumption					
*Yes*	30.8 (97)	53.9	3.639	1	0.056
*No*	69.2 (218)	42.3			

Single mothers’ educational background varied, but one in three of single mothers in the sample (36.2%) had University education. Regarding employment status, as much as 73.4% of the participants held full-time jobs, and despite that, 79.7% of the sample reported economic hardship to meet their daily expenses during the last 12 months. This was mainly due to the fact that more than half of the participants (55.0%) reported monthly family income less than €1500, and a further 27.8% less than €1000.

Regarding support from the state, the 40.8% of the sample have reported that they were not single mothers’ allowance recipients, and nearly half of them (48.1%) did not receive financial support from the father of their child/children.

Regarding the participants’ health status, 49.4% of the single mothers have reported a long-standing illness, and depression was the most commonly reported one with the rate of 32.2%.

### Prevalence of mental distress in relation to socio-demographic characteristics

As many as 44.6% of the sample appeared to experience clinically significant symptoms of mental distress (GHQ-28 total score ≥ 5). Table 1 also presents the observed differences in mental distress according to socio-demographic characteristics of the participants. The most pronounced statistically significant differences in the prevalence of mental distress as measured by the GHQ-28 were observed for age, educational level, monthly family income, economic hardship and the presence of a long-standing illness. With a prevalence of mental distress as high as 57.1%, single mothers in the age group 35–44 seem to experience the highest levels of mental distress compared to both the younger age group (40.5%) as well as the older age group (46.5%); x^2^ = 6.417, df = 2, *p* = 0.040.

The prevalence of mental distress was statistically significantly higher among single mothers who had higher education (63.5%) and junior high school education (60.0%), compared to those who had university education (40.7%, x^2^ = 10.29, df = 3, *p* = 0.016). A stepwise, association was also observed in terms of decreasing monthly family income, with an observed prevalence of mental distress as high as 65.5% among those with monthly family income less than 1000 euros versus 21.4% among those with income higher than 3500 euros/month (x^2^ = 15.29, df = 5, *p* = 0.009).

Among single mothers who reported economic hardship for daily expenses in the last 12 months, the prevalence mental distress appeared twice higher than those who did not report economic hardship (57.2% vs 23.4%, x^2^ = 23.23, df = 1, p = < 0.001). Similarly, and not surprisingly, statistically significant higher prevalence of mental distress was observed among those reporting long standing illness (65.2% vs 35.8%, x^2^ = 26.97, df = 1, *p* = < 0.001).

No statistical associations were observed among other variables examined, such as single-family status, the age of their child/ren, whether they were members of the SMA or if they are recipients of single mothers’ allowance or financial child support from the father. Furthermore, even though not statistically significant at the 5% level the prevalence of mental distress appeared elevated among the economically inactive group compared to mothers who reported working full-time, as well as among mothers who have been in their current status for shorter period of time.

The results of the backward stepwise logistic regression analysis are presented in Table 2. Starting with thirteen socio-demographic variables, the final predictive model included five variables. The strongest negative association was observed with regards to monthly family income. There was a clear stepwise increase in the likelihood for psychophysical distress among single mothers across decreasing levels of income. In fact, mothers in the lowest income category were more than 6 times as likely to report mental distress compared to mothers in the higher income category (OR = 6.658 95% CI: 1620-27,495). A statistically significant association was also observed in terms of house tenure (used here as an additional socio-economic position indicator) with single mothers residing in rented accommodation appearing at least twice as likely to experience psychophysical distress (OR = 2.163 95%, CI: 1.106–4449) compared to mothers who own their house.

**Table 2:** Adjusted odds ratios (and 95% CI) of clinically significant psychological distress (GHQ-28 ≥ 5) by personal, family, economic and health behavior characteristics as estimated in multivariable backward stepwise logistic regression analysis

GHQ-28 (≥5/6) Psychological distress	Adjusted^a^ OR (95% CI)	*P* value
Monthly family income		
> 3500	1	0.202
2500–3499	2,619 (0.598–11.478)	0.143
2000–2499	3.121 (0.680–14.323)	0.190
1500–1999	2.609 (0.622–10.947)	0.058
1000–1499	3.839 (0.954–15.446)	**0.009**
< €1000	6.658 (1.612–27.495)	
Age category		
23–34	1	
35–44	3447 (1780-6673)	**< 0,001**
≥ 45	2787 (1258-6176)	**0,012**
Duration in single mother status		
≥ 5 years	1	
2–4 years	0,705 (0,402-1238)	0,224
< 2 years	2272 (1116-4626)	**0,024**
House tenure		
Owned	1	
Rented	2163 (1106-4230)	**0,024**
Physical Activity		
No	1	
Yes	2152 (1264-3663)	**0,005**

^a^Factors included in the first stage: age, educational level, employment status, single motherhood status, duration as single mother, number and age of independent child/ren, monthly family income, single mothers’ allowance, financial support from the father, member of the Single Mothers’ Association, smoking status, alcohol consumption, physical activity and house tenure

In addition, there appeared to be a statistically significant association with age. Single mothers between 35 and 44 years old, were more than three times more likely to experiences mental distress, compared with the younger age group (OR = 3.46, 95% CI: 1780-6673). The same pattern, but to a lesser degree, applies to the single mothers of ≥45 years of age (OR = 2.787, 95% CI: 1.258–6176). A statistically significant association was also observed in terms of the period in a single-parent family Specifically, it was observed that single mothers who have been in their current status for less than 2 years, experience the highest level of mental distress as they were twice as likely to score high on the GHQ-28 compared to those who have been single mothers for longer than 5 years (OR = 2.272, 95% CI: 1.116–4626).

Finally, even though somewhat surprising, a statistically significant association was observed in terms of physical activity, with mothers who report been more physically active appearing more likely to experience mental distress. Results were unchanged when using the forward method instead.

### Perceived social support among single mothers and association with mental distress

In terms of the perceived social support, taking into consideration the theoretical range of the scale which is 24–96, the minimum and maximum SPS scores that have been observed based on the participants’ responses were 42–96. Therefore, these observed values were distributed into quartiles for statistical analysis purposes. Based on that, the following socio-demographic characteristics found to be significantly related with the lower levels of perceived social support: *lower monthly family income*, *economic difficulties and therefore being a recipient of single mother allowance* (*p* < 0.01), *the family status as being unmarried mothers* compared to those who were divorced, separated or widows (*p* = 0.007). Apart from that, single mothers who were registered member of the SMA seemed to have higher levels of perceived social support (*p* = 0.009).

The prevalence and odds ratios of mental distress as measured by the GHQ-28 across quartiles of the levels of SPS before and after adjusting for all socio-demographic variables are shown on Table 3. These results reveal an association with mental distress and social support.

**Table 3:** Prevalence and odds ratios (95%CI) of psychological distress across decreasing levels of Social Provision (SPS) as estimated before and after adjusting for socio-demographic variables in a hierarchical four-step multivariable logistic regression model

	Prevalence of psychological distress (GHQ-28 ≥ 5)	Unadjusted OR (95% CI) *P* value	Fully Adjusted ^a^ Model IV OR (95% CI) *P* value
T4: Higher quartile of perceived social support (values: 87–96)	30.5%	1	1
Τ3 (values:78–86)	44.0%	1692 (0,882-3246) 0,113	1535 (0,657-3564) 0,322
Τ2 (values:71–77)	49.4%	**2208** (1167-4175) 0,015	**3278** (1436-7481) 0,005
Τ1: Lower quartile of perceived social support (values:42–70)	78.4%	**7808** (3789-16,088) 0,000	**7930** (3087-20,371) 0,000

^a^The fully adjusted model presented above includes the following variables single family type, monthly family income, single mother allowance recipient, financial support from the father, member of SMA, all health related behaviors, age, educational status, number and age of children, employment status, economic difficulties, presence of long standing illness

The prevalence of mental distress displayed a clear stepwise increase across quartiles of decreasing levels of perceived social support with as many as 78.4% of mothers scoring five or higher on the GHQ-28 in the quartile with the lowest levels of social support compared to 30.5% among mothers reporting the highest levels of social support. In the logistic regression model, the likelihood of reporting mental distress was estimated at nearly eight times higher among the quartile of participants with the lowest levels of social support (OR = 7808, 95% CI: 3789 – 16,088), and more than twice higher among those in the second quartile (OR = 3278, 95% CI: 1436-7481). After adjusting for the potential confounding effect of socio-demographic variables, this association did not attenuate in the fully adjusted model (OR = 7930, 95% CI: 3087-20,371).

As shown in Table 4 below, a similar pattern of negative association between social support and mental distress is largely observed across all four subscales of the GHQ-28 (Somatic symptoms, Anxiety and Insomnia, Social disfunction and Severe depression). All associations were statistically significant (*p* < 0.001), with the strongest correlations observed for the sub-scales of severe depression and social dysfunction.

**Table 4:** Association between GHQ-28 sub-scale scores with decreasing level of Social Provision (SPS)

Subscale	GHQ A Somatic symptoms		GHQ B Anxiety and Insomnia		GHQ C Social dysfunction		GHQ D Severe depression	
	Mean	S.D.	Mean	S.D.	Mean	S.D.	Mean	S.D.
T1	1077	4744	1265	5438	1099	4146	742	4874
T2	724	4160	853	4758	816	2472	343	3823
T3	727	4323	884	5944	743	3239	344	4406
T4	737	5010	737	5438	630	3169	160	2666
Overall	811	4784	927	5716	817	3689	389	4497
*P* value	**< 0,001**		**< 0,001**		**< 0,001**		**< 0,001**	
Pearson	−0.260		−0.325		−0.451		−0.471	
Spearman’s rho	−0.248		−0.323		−0.420		−0.470	

## Discussion

### Main findings

The aim of the present study was to obtain an estimate of the prevalence of mental distress experiences among single mothers in Cyprus, as measured by the GHQ-28, as well as association with sociodemographic characteristics and perceived social support. Previous studies tend to use specific screening tools for clinical depression and anxiety [18–20], whereas the GHQ-28 is a widely used non-specific measure of pre-clinical mental distress in community sample [39]. The added benefit of using this measure rather than the measures used in previous studies, is based on the fact that its four subscales, seem to be a good match with the variables, that based on the literature single mothers experience in a higher extent as a result of their family situation [35]. Nearly half of the single mothers in our study reported mental distress based on a cut-off point of 5/6 set on the GHQ-28. Even though no direct cross-national comparisons can be made since to the best of our knowledge no other studies have used the GHQ-28 to assess the mental distress of single mothers, this outcome is alarming. Of course, the expected elevated mental distress experience by single mothers described here for the first time among single mothers in Cyprus, is not surprising based on the international literature focusing on similar constructs and using other tools [9, 10, 20, 30, 49]. There is evidence in the literature to support that single mothers compared to any other group of women, experience greater levels of mental distress [9, 29], physical disability [16], poorer psychological health [20] and lower levels of life satisfaction and happiness [50]. Overall, the literature describes elevated levels of depressive and anxiety symptoms and psychosomatic illness among single mothers irrespective of the sociopolitical and welfare system across different countries. As they point out, it is likely that besides socioeconomic status and social support, factors such as time pressure, lower-grade jobs, reduced usage of social support, biographical events, personality traits and social marginalization, might also influence the health of single mothers.

The socioeconomic factors investigated in this study, point to both similarities and differences of single mothers in Cyprus compared to other countries. Participants in our study, had a mean age of 39 years old and have high educational attainment. As many as 36% had university education which compares to census official estimates among general female population aged 20–49 (31%). A large proportion held a full time job (73.4%) and 8.7% were economically inactive, which is much lower than official census statistics among women aged 20–49 in the general population (20%). Older age, high educational attainment and high economically active status among Cypriot single mothers, come in contrast to previous published studies [16, 24, 27, 29]. On the other hand, our findings are consistent with previous studies with regards to the relatively higher economic disadvantage experienced by single mothers. Several studies have reported that like in the present study single mothers generally have lower income, and experience financial hardship with daily expenses [10, 51, 52].

### Sociodemographic correlates with mental distress

The analysis revealed strong associations between sociodemographic and socioeconomic factors and psychological well-being, indicating in particular that mothers in the 40s and mothers in the first 2 years after divorce or separation were prone to mental distress. These associations remained statistically significant in multivariable analyses. Previous studies have shown, that relative to other women, younger single mothers were twice as likely and mid-age single mothers were four times as likely, to experience higher levels of stress [20]. Similar results were identified in the study of Sperlich et al., as perceived stress was elevated in single mothers in the age group of 30–39 years [30]. However, several studies have also shown that the frequency of depressive symptoms due to chronic stress among single mothers, seems to be increased at younger ages [16, 30]. The association with the age can be linked with two related variables investigated in this study that correlate with increased prevalence of mental distress: the first years after divorce or separation and having children younger than 12 years old. Therefore, it could be hypothesized that single mothers in the mid-thirties and early forties, might have been single parents for shorter period of time [53], or have children less than 12 years old [54].. Significant variations in regards to the duration of single parent families, are also observed in the present study: those who were single between 1 and 2 years were more likely to suffer mental distress compared to those who were in a single parent family for less than a year were or more than 5 years. The above finding, according to relevant studies, is probably due to: either the marital relationship was problematic and therefore its dissolution was a relieve by any possible frictions in the first year [53, 55, 56] or that the problems associated with a single parent family become more perceptible as they accumulate and are more likely to escalate after the first year [55].

### Association of education, employment and financial hardship with mental distress

The strongest and most consistent associations with mental distress was with monthly family income and financial hardship. There was clear evidence of a social gradient, with single mothers who reported lower monthly family income were up to seven times more likely to experience mental distress. The association between the relatively poorer psychological health of single mothers and economic status is also in agreement with the findings of previous studies [11, 27, 30, 57].

With regards to educational attainment, an inverse gradient was also observed with single mothers with a higher educational attainment perceiving more mental distress. This could be mainly attributed to increased personal and family demands, such as child-related and work-related burdens. Studies have reported that the greater distress observed among single mothers could be completely explained by their greater exposure than partnered mothers to financial strain, psychosocial work stress, and work-family conflict [29].

In the present study, employment status also seems to be associated with mental distress. Economically inactive single mothers and those who held part-time jobs seem to experience higher levels of mental distress. Even though, the cross-sectional nature of this study, does not allow to infer causality in the observed relationship between employment status and health. However, although studies show that employed single mothers report significantly higher levels of mental distress compared to their married counterparts [29], it should be noted that there is an added complexity in the relationship between single-mother status and employment. Literature supports, that there are several variations in the correlations between the employment status and health. This is mainly due to the fact that the relationship between single parent households and the labor market is affected by a number of factors, such as labor market opportunities, social infrastructure, social welfare, individual welfare benefits, the degree of support from the extended family and the tax system [58].

Work increases monthly family income and restricts economic hardship, thus it might alleviate the additional economic-related stress [2, 19], as well as provide additional opportunities for social support. Socialization through work is considered to exert a protective effect for the well-being of single mothers [50] as it has been argued that distress due to loneliness decreases with increasing working time, and this may explain in part our study results.

Despite that, there is evidence to suggest that even though single mothers are more likely to be employed than any other group of women [50, 57], they hold positions with lower earnings, or by necessity, are working part-time as they do not have the necessary support for the care of their children. This is also strongly apparent in the present study, since despite the fact that the great majority of the sample has high educational level (59.7% higher/University education) and is working full-time (73.7%), the majority reported their monthly family income to be less than 1500 euro (55.0%). The difficult financial situation of single mothers in our sample is further highlighted by the fact that as many as 40.8% are not recipients of the single motherhood allowance. The strong association between mental distress and single mothers in the lowest income category observed in this study, is an important finding, which may reflect deficiencies in the Cyprus social welfare system. Therefore, a better financial support system is required which will be based on income criteria and family status, as there is evidence to suggest that single family allowances can significantly reduce poverty [58].

### Association of social support with mental distress

A strong and independent association between socio-economic circumstances, mental distress and Perceived lack of social support was observed among single mothers. This finding is consistent with previous studies [16]. Emotional support provided by social ties enhance the psychological well-being. In turn, this may reduce the risk of unhealthy behaviors and poor physical and psychological health (Malik AA: The study of social support as a determining factor in depressed and non-depressed as measured by an indigenously developed social support scale. Unpublished PhD Dissertation). The consequences of decreased social support results in mental distress, whereas, supportive relationships can reduce the likelihood of experiencing depression [1, 2].

According to Cutrona & Russell [43], social support may take the form of *tangible support* (e.g. financial assistance, care in minding) and *emotional support*. Their importance however may vary depending on the current needs of the individual, and might be more effective for creating coping mechanisms to deal with stressful situations, if this corresponds to the factors that creates the stress. In the context of this study, we used the overall perceived levels of social support, and we did not evaluated the sources or type of social support. Thus, while it is not possible to investigate in more detail the particular needs of single mothers in terms of social support needed by single mothers, the factors more strongly associated with mental distress are of economic nature. In fact, there was evidence to suggest that financial status was strongly related to lack of social support. Specifically, single mothers with monthly family income less than 1000 euro, allowance recipients, experiencing financial difficulties and those in rented house reported the lowest level of perceived social support (results not shown in detail). This not only supports the likely importance of tangible support for single mothers over and above emotional support but it also highlights the inadequate social welfare structures and policies in Cyprus,

These findings are in consistency with the majority of related studies [21, 23, 36, 50, 59–61]. Beyond the fact that financial security provides single mothers with the ability to provide their family with the necessary for living, it is also an important parameter in providing them with opportunity for socializing. Apart from the financial distress, lack of tangible support may result in lack of free time, and thus reduced opportunities for single mothers to dedicate time to themselves [62, 63], which may also adversely affect their psychological well-being.

Another important issue discussed in the literature is the potentially supportive role of older children as a likely source of emotional support for single mothers. There aren’t many studies in the international literature that have tried to distinguish the potential differential health status among single-mothers depending the age of their children. Even though older children might constitute an important factor for supporting their single mothers compared to children of younger age [54], or those with older children might have more opportunities for employment and the ability to effectively combine work with family responsibilities (work-family conflict), which can be a protective factor for their psychological well-being [50, 54, 64], this might be the case of ages older than 12 years. However, in this study no statistically significant differences were observed.

### Strengths and limitations

The results of this study should be considered in light of some strengths and limitations. This is the first study to the best to our knowledge, describing the psychological well-being among single mothers in Cyprus as well as the lack of social support as a determinant of mental distress. Furthermore, while several studies in the international literature focused on clinical entities, such as depression and anxiety, not many studies have used a generic tool of symptoms of psychopathology among single-mothers. Through this study, the uneven distribution of socioeconomic and psychosocial recourses and the impact this may have on vulnerable population groups, such as single mothers is highlighted to a great extent. There are also a number of limitations. First, due to the cross-sectional nature of the study, the directionality of observed relationships cannot be ascertained. Thus, while we cannot draw conclusions about causality (i.e. financial difficulties impacting on psychological well-being), it is reasonably likely to assume that social and economic circumstances act as a source of stress and thus increase the risk of mental distress among single mothers rather than the other way around [30]. Additionally, as it was a selective sampling, the findings may not be generalisable to all single mothers in Cyprus. Second, we recognize that this study does not include a control group of partnered mothers. However, this is the first study conducted in Cyprus concerning the assessment of mental health of single mothers and correlations with Social Support and the extent of social gradient within this vulnerable group. Third, there is no information with regards to some potentially important variables, for example the quality of the couple relationship prior to divorce or separation. For example, the experience of marital violence is well known to be significantly associated with mental distress. Finally, a longitudinal investigation with repeated measures over time is more suited in order to better understand some of the observed associations. For example, while in this study it appeared that the shorter the duration of single mother status the higher the mental distress, this finding is based on a cross-sectional comparison of a different set of mothers, based on the number of years since their divorce or separation rather than based on an investigation into the trajectory of mental well-being of single mothers followed through time.

## Conclusion

This study highlights that as many as half single mothers in Cyprus experience clinically significant symptoms of mental distress. Financial hardship and lack of social support are some of the primary determinants. With a steady rise in the proportion of single-parent families headed by a mother both in Cyprus as well as all over Europe, these findings highlight a significant issue that would adversely affect many women and consequently their children and the community as a whole.

The need to further elucidate the social, economic and psychosocial factors that adversely or positively determine the psychological well-being of single mothers is important in order to develop ***community support systems*** and to adopt appropriate ***social welfare policies reform*** [65]. Additionally, the determination of these factors will be useful in order to design more ***targeted prevention programs***, such as group sessions targeting single mothers, which found to be helpful in decreasing depressive symptoms, negative thinking, and chronic stressors. Some good examples could be “the cognitive-behavioral group intervention” [66], the “behavioral interventions or parenting programs” proposed by Taylor and Conger [67], and the “parental training program” developed by Weihrauch [68].

## Abbreviations

GHQ-28: General Health Questionnaire – 28 items
SMA: Single Mothers’ Association
SPS: Social Provision Scale
